# Associations between parent–child outdoor visits and preschool-aged children’s screen time: a cross-sectional study

**DOI:** 10.1186/s44167-025-00089-5

**Published:** 2025-11-06

**Authors:** Henna Launistola, Reetta Lehto, Elina Engberg, Josefine Björkqvist, Jenna Rahkola, Nina Simonsen, Nanna Wackström, Emmi Tilli, Eva Roos, Carola Ray

**Affiliations:** 1 Folkhälsan Research Center, Topeliuksenkatu 20, 00250 Helsinki, Finland; 2 Faculty of Medicine, University of Helsinki, P.O. Box 63, 00014 Helsinki, Finland; 3 Department of Food and Nutrition, University of Helsinki, P.O. Box 66, 00014 Helsinki, Finland; 4 Institute of Applied Health Sciences, University of Aberdeen, Foresterhill, Aberdeen, AB25 2ZD UK; 5 Department of Public Health, University of Helsinki, P.O. Box 20, 00014 Helsinki, Finland

**Keywords:** Early childhood, Preschool children, Screen time, Outdoor play, Outdoor space

## Abstract

**Background:**

Children’s decreasing outdoor time and increasing screen time (ST) raise concern, as both can impact children’s health unfavourably. However, little is known about the association between young children’s context-specific outdoor time and ST. This study investigated the associations between parent–child outdoor visit frequency in nature, own yard, playground, and in total and preschoolers’ ST on weekdays and weekends.

**Methods:**

Data were collected via a survey assessing parent–child outdoor visits and a 7-day ST diary completed by parents of children (*n* = 673, 3–6 years) participating in the DAGIS intervention study in Finland. The cross-sectional associations were examined by linear regression analyses adjusted for child age, sex, socioeconomic factors, and season.

**Results:**

More frequent parent–child visits to own yard (B −1.98, 95%CI −3.43, −0.52) and nature (B −2.31, 95%CI −4.01, −0.60) were associated with less weekday ST among children, whereas more frequent visits to playground (B −3.68, 95%CI −7.18, −0.18) were associated with less weekend ST. Also, more frequent visits to nature (B −2.28, 95%CI −4.20, −0.38), own yard (B −2.38, 95%CI −4.03, −0.73), and playground (B −2.48, 95%CI −4.71, −0.25) were associated with less whole-week ST. More frequent total outdoor visits were associated with less weekday (B −1.547, 95%CI −2.38, −0.71), weekend (B −1.91, 95%CI −3.38, −0.45), and whole-week ST (B −1.77, 95%CI −2.71, −0.84).

**Conclusion:**

In conclusion, visiting different outdoor spaces was associated differentially with children’s ST on weekdays and weekend days, whereas total outdoor visit frequency was associated with less ST throughout the week. Hence, municipalities should ensure the availability of different types of safe outdoor spaces in neighbourhoods to provide alternatives for young children’s screen use.

**Trial registration number:**

ISRCTN57165350, Registration date 08/01/2015.

**Supplementary Information:**

The online version contains supplementary material available at 10.1186/s44167-025-00089-5.

## Introduction

Before the era of screen devices, outdoor free play was one of the most common and preferred ways of spending time for children across different age groups [1]. However, environmental change, urbanization, and digitalization are changing people’s daily lives at an accelerating pace. Using screens has become a part of our cultural and social environment [2], and spending time on screen devices, such as mobile phones, tablets, TVs, and computers, is now a common sedentary behaviour also among the youngest of children [3, 4]. Indeed, according to studies mainly conducted in Western countries, screen use has increased, whereas outdoor play has decreased [5–9]. However, screen use and outdoor time do not necessarily exclude each other [2], and both behaviours are inarguably part of children’s lives today.

This study focuses on children aged 3 to 6 years. For 3–4-year-old children, the World Health Organization’s screen time (ST) recommendation is a maximum of one hour per day [10], and for 5-year-olds limiting the time spent sedentary, especially on screens, is advised [11]. However, young children’s ST is often higher than recommended. In Finland, screen use of 4–6-year-olds is one hour or more in 44% on weekdays and 81% on weekends or holidays [12]. In Europe, 50% and globally 42% of 3–4-year-olds meet the one-hour ST recommendation [13]. The high amount of ST is concerning, as it associates unfavourably with young children’s health outcomes such as overweight and obesity [14], poorer sleep outcomes [15, 16], and near-sightedness [17]. It is also associated with psychosocial symptoms [18] and poorer cognitive and language development [19, 20].

Spending time outdoors, on the other hand, has many benefits for preschool-aged children, as it is associated with, for instance, more physical activity [15, 21–23], better sleep quality [21, 24], lower perceived stress, and healthy gut microbiota [25]. The positive effects also include better motor and cognitive development, better social and emotional outcomes [26], and among schoolchildren also delayed onset of near-sightedness [27]. Being outdoors and in nature is also associated with a greater connection to nature among preschoolers [28], which benefits children’s health [29].

Children’s outdoor time can take place in home, at school or preschool, in nature, and in the community [30]. Outside of preschool, contexts for young children’s outdoor play can be both public spaces, such as parks, streets, and playgrounds, and private spaces, such as gardens and own yards [12, 31]. Nature, often including forests, beaches, and other natural environments, is also a relatively common outdoor space for families to visit [12, 28, 32, 33]. However, young children are often dependent on their parents to visit outdoor spaces, whereas older children can visit them more independently, even in cities. In addition, parental safety concerns may also a be a factor determining to what extent children are permitted to spend time outdoors [34–36].

The associations between outdoor play environment and children’s health and behaviour are often examined with regard to physical activity [37] or sedentary time [38], but relatively few studies have investigated context-specific outdoor time and preschool-aged children’s ST. Moreover, studies examining young children’s ST and outdoor time have shown mixed results. In a Canadian study, outdoor time was not associated with 3–6-year-old children’s ST [22]. Similarly, no associations were found between preschool-aged children’s outdoor play and TV-viewing [39, 40] or weekly ST among Australian 3–5-year-olds [41]. However, an Indian study with 3–15-year-old children noted that lack of outdoor play was associated with excessive (>2 h/day) ST [42], although it is important to acknowledge that the study sample was not restricted to preschool-aged children. Moreover, in longitudinal studies, playing outdoors in early childhood predicted less computer use and gaming in later childhood [43], and more ST at 3–4 years was associated with less outdoor play at 10–11 years [7]. Furthermore, a systematic review concluded that there is insufficient evidence of an association between park visits and ST in under 5-year-olds [44].

In addition, some studies have investigated how neighbourhood physical environments associate with children’s ST. In one study, preschool-aged children living further away from green or open spaces had more weekly TV time than those living closer to these spaces [45]. In another study, greater neighbourhood greenness was associated with less TV-viewing on weekends for 4–5-year-old boys but not girls [46]. In addition to environmental factors, also socioeconomic factors have been found to be linked to both children’s ST [44] and outdoor time [7, 47].

More detailed knowledge about young children’s screen use and outdoor time is warranted as it can inform health promotion strategies and policies that aim to influence these behaviours. Understanding the relationship between the two behaviours can also help design interventions aiming to reduce children’s ST through outdoor activities. As earlier research has focused mainly on school-aged children and overall outdoor time, environmental characteristics, and physical activity, this study aimed to investigate how context-specific outdoor visits, including visits to own yard, nature, and playground, during leisure time are associated with preschool-aged children’s ST on weekdays and weekends.

## Methods

### Participants

Data used in this study comprise cross-sectional survey data collected during baseline measurements in a clustered randomised controlled trial DAGIS in September–November 2017. The participants were Finnish children aged 3–6 years (*n* = 801) in two municipalities in Southern Finland attending the early childhood education and care (ECEC) centres, and their parents. Families were invited via ECEC centres (*n* = 32), and parents gave informed consent on behalf of themselves and their child prior to data collection (participation rate 47%) [48]. The DAGIS intervention aimed to promote healthy eating habits, physical activity, and self-regulation skills, and to decrease ST among children. Ethical approval was granted for the DAGIS study by the Research Ethics Committee of the University of Helsinki (22/2017; 16 May 2017). More information about the DAGIS study can be found elsewhere [48].

### Measurements

*Child sedentary screen time* Children’s ST was assessed with a parent-reported 7-day ST diary. Parents were asked how much time their child spent sedentary using screens (if duration over 10 min) each day outside ECEC hours. The diary was modified from a previous validated diary [49] by adding portable screens as an answering option. Time (h and min) spent in different screen activities (watching TV or DVDs/videos or using tablets, smartphones, and computer/play consoles) was asked separately and summed to get the daily total ST. Average weekday and weekend day ST (min/day) was calculated if data were available from at least 3 weekdays and 1 weekend day, respectively. From these variables, the average weighted whole-week ST (min/day) was calculated (weekday average x 5 + weekend day average x 2, divided by 7) if the 4-day criterion was fulfilled.

*Parent–child outdoor visits* Frequency of parent–child nature, own yard, and playground visits was enquired from the parent with a question developed for the DAGIS study. The question was “How often during the last month has your child visited the following places with at least one adult in the family? A. Nature/forest, B. Own yard, C. Park/playground”, the response options being “not at all”, “1–3 times/month”, “1–2 times/week”, “3–6 times/week”, and “daily”. In the item “C. Park/playground”, the two statements were combined to avoid overreporting, as they both have a similar meaning in Finnish language, both referring to an outdoor recreational space outside one’s own yard. For the analyses, the response categories were recoded into mean values of each category (0, 0.5, 1.5, 4.5, and 7 times/week), and the variables were used as continuous variables. To examine the total parent–child outdoor visit frequency, a continuous sum variable was formed from the recoded variables for nature, own yard, and playground visit frequencies.

Child’s age was calculated from the parent-reported date of birth, and child’s sex was enquired in the parents’ survey (boy/ girl/ I don’t want to answer). Highest parental educational level (PEL) and relative household income were used as indicators for socioeconomic status (SES). Highest PEL in the family was measured by asking responding parents their highest education level and also that of the other parent if the child lived with both parents (7 response options from comprehensive school to doctoral/licentiate degree), and the highest PEL in each family was selected. PEL was recategorized into low (comprehensive school education or upper secondary education), middle (Bachelor’s degree), and high educational level (Master’s degree or higher). Relative household income was determined from the parent-reported monthly household income (10 response options ranging from < 500€ to > 10 000€ per month). The income was then divided by the number of persons in the household (weighted for adults and children). The variable was transformed into a continuous variable from the midpoint of each category.

In addition, the questionnaire completion date was used as an indicator for season. The questionnaires were filled in between September and November 2017. The response dates were recoded into two categories: (1) September and (2) October-November. October and November were combined as the number of respondents in November was remarkably small (*n* = 13), and the majority responded during the first week of November. In addition, in Finland, September is the first autumn month, but it is still a warmer season with more daylight hours, providing good opportunities for outdoor activities. In October and November, by contrast, the weather is usually colder and rainier, and the daylight hours decrease rapidly.

### Statistical analyses

Descriptive results are reported as frequencies and percentages or mean values and standard deviations. Correlations were calculated with Spearman’s Rho (Supplementary Material 1). To examine associations, multiple multivariate linear regression analyses were conducted, in which children’s ST was the dependent variable and parent–child nature, playground, and own yard as well as total outdoor visit frequencies were the independent variables. Out of the 801 parents that gave their consent to participate in the study, a total of 673 parents filled in both the ST diary (so that data were available from at least 3 weekdays and/or 1 weekend day) and answered the outdoor visit frequency question (a response to at least one of the question items). Data from these 673 participants was used in the analyses. Three different regression models were used: (1) unadjusted, (2) adjusted for child age and sex, and (3) adjusted for child age and sex, PEL, relative household income, and season. In the regression analyses only the respondents who had data on all variables included in the respective models were included in the analyses. The covariates used in the analysis were based on previous literature [7,  50–52]. The linear regression analyses were run separately for each independent variable. The level of significance was set at *p* < 0.05, and the 95% confidence interval was used. Statistical analyses were conducted with IBM SPSS Statistics (version 29.0).

## Results

### Descriptive results

Table 1 presents the descriptive results. Among the 673 children that had sufficient data on ST and outdoor visit frequency, the mean age was 5.2 years (SD 1.0) and 46% were girls. Nearly half of the families (45%) belonged to the middle category of the PEL categorization. Most of the parents (64%) filled in the questionnaire in September.

Children’s average ST was 70 min/day on weekdays, 125 min/day on weekend days, and 86 min/day for the whole week. During the previous month, the most common parent–child visit frequencies were daily in own yard (44% of respondents) and 1–3 times a month in both nature and playground (37% and 35%, respectively). The proportions of families not visiting own yard, nature, or playground at all during the last month were 1%, 4%, and 17%, respectively.

**Table 1:** Descriptive results of study variables for participants (*n* = 673)

Variable		*n*	%	Mean (SD)
Respondent	Mother	609	90.8	
	Father	58	8.6	
	Other	4	0.6	
Child’s age	Age (years)	673		5.2 (1.0)
Child’s sex	Female	307	45.6	
	Male	366	54.4	
Highest PEL^a^ in the family	Low	213	31.7	
	Middle	302	44.9	
	High	157	23.4	
Relative household income	Euros/month	610		1930.9 (775.6)
Response month	September	425	64.6	
	October-November	233	35.4	
Children’s screen time	Weekday (min/day)	660		70.0 (39.9)
	Weekend (min/day)	635		124.6 (68.2)
	Whole week (min/day)	622		85.8 (43.6)
Parent–child nature visits	Not at all	29	4.3	
	1–3 times a month	248	36.8	
	1–2 times a week	239	35.5	
	3–6 times a week	123	18.3	
	Daily	34	5.1	
	Continuous (times/week)	673		1.9 (1.9)
Parent–child own yard visits	Not at all	9	1.3	
	1–3 times a month	36	5.3	
	1–2 times a week	94	14.0	
	3–6 times a week	235	34.9	
	Daily	299	44.4	
	Continuous (times/week)	673		4.9 (2.2)
Parent–child playground visits	Not at all	110	16.3	
	1–3 times a month	237	35.2	
	1–2 times a week	219	32.5	
	3–6 times a week	86	12.8	
	Daily	21	3.1	
	Continuous (times/week)	673		1.5 (1.7)
Parent–child total outdoor visits	Times/week	673		8.3 (3.9)

^a^Parental education level: low = comprehensive school or upper secondary education, middle = Bachelor’s degree, high = Master’s degree or higher

### Associations between parent–child outdoor visits and child ST

Table 2 shows the results of the linear regression analyses run separately for each outdoor space and each model.

#### Weekday screen time

In the unadjusted (model 1) and the child’s age- and sex-adjusted (model 2) analyses, more frequent visits to nature, own yard, playground, and outdoors in total were all negatively associated with children’s weekday ST. However, in the analyses fully adjusted also for season and SES (model 3), only visits to own yard (B −1.98, 95% CI −3.43, −0.52), nature (B −2.31, 95% CI −4.01, −0.60), and outdoors in total (B −1.547, 95% CI −2.38, −0.71) remained significantly negatively associated with weekday ST.

#### Weekend day screen time

More frequent parent–child playground visits and total outdoor visits were negatively associated with children’s weekend ST in all linear regression models. Results in the fully adjusted models were significant for playground visits (B −3.68, 95% CI −7.18, −0.18) and for total outdoor visits (B −1.91, 95% CI −3.38, −0.45). Frequency of visits to nature or own yard were not associated with children’s weekend ST.

#### Whole-week screen time

In all linear regression models, a significant negative association was found between child whole-week ST and own yard, nature, playground, and total outdoor visit frequency. In the fully adjusted model, more frequent visits to own yard (B −2.38, 95% CI −4.03, −0.73), nature (B −2.28, 95% CI −4.20, −0.38), and playground (B −2.48, 95% CI −4.71, −0.25) and higher total outdoor visit frequency (B −1.77, 95% CI −2.71, −0.84) were negatively associated with children’s whole-week ST.

**Table 2:** Linear regression results for parent–child context-specific outdoor visits and children’s screen time (*n* = 554–660)

	Children’s screen time (min/day)															
Model	Frequency of visits	Weekday					Weekend day					Whole week				
		95% CI				adj. *R*^2^	95% CI				adj. *R*^2^	95% CI				adj. *R*^2^
		B	Lowest	Highest	*p*		B	Lowest	Highest	*p*		B	Lowest	Highest	*p*	
1	Nature	−2.22	−3.86	−0.59	**0.008**	0.01	−1.33	−4.18	1.52	0.360	0.00	−1.98	−3.83	−0.13	**0.036**	0.01
	Own yard	−2.04	−3.41	−0.67	**0.004**	0.01	−2.14	-4.54	0.26	0.080	0.00	−2.37	−3.92	−0.81	**0.003**	0.01
	Playground	−2.42	−4.23	−0.62	**0.009**	0.01	−5.16	−8.32	−2.00	**0.001**	0.01	−3.38	−5.42	−1.35	**0.001**	0.02
	Total	−1.61	−2.38	−0.84	**< 0.001**	0.02	−1.94	−3.29	−0.58	**0.005**	0.01	−1.84	−2.72	−0.97	**< 0.001**	0.03
2	Nature	−2.18	−3.79	−0.56	**0.008**	0.04	−1.05	−3.85	1.74	0.460	0.04	−1.85	−3.66	−0.04	**0.046**	0.05
	Own yard	−2.19	−3.54	−0.83	**0.002**	0.05	−2.12	−4.49	0.25	0.079	0.05	−2.40	−3.94	−0.87	**0.002**	0.06
	Playground	−2.10	−3.89	−0.30	**0.022**	0.04	−4.16	−7.29	−1.03	**0.009**	0.05	−2.83	−4.84	−0.81	**0.006**	0.05
	Total	−1.60	−2.37	−0.83	**< 0.001**	0.05	−1.70	−3.04	−0.35	**0.013**	0.05	−1.74	−2.61	−0.87	**< 0.001**	0.07
3	Nature	−2.31	−4.02	−0.60	**0.008**	0.07	−1.99	−4.98	1.01	0.193	0.06	−2.28	−4.20	−0.36	**0.020**	0.08
	Own yard	−1.98	−3.43	−0.52	**0.008**	0.07	−2.39	−4.97	0.18	0.069	0.06	−2.38	−4.03	−0.73	**0.005**	0.09
	Playground	−1.94	−3.95	0.07	0.058	0.06	−3.68	−7.18	−0.18	**0.039**	0.07	−2.48	−4.71	−0.25	**0.030**	0.08
	Total	−1.55	−2.38	−0.71	**< 0.001**	0.08	−1.91	−3.38	−0.45	**0.011**	0.07	−1.77	−2.71	−0.84	**< 0.001**	0.10

Analyses were run separately for each outdoor space. Statistically significant p-values are bolded

Models:

(1) Unadjusted

(2) Adjusted for child age and sex

(3) Adjusted for child age and sex, PEL, relative income, and season

## Discussion

This study investigated the cross-sectional associations between parent–child outdoor visit frequency and preschool children’s sedentary ST on weekdays, on weekend days, and during the whole week. The results showed that more frequent parent–child visits to nature and own yard were associated with less ST on weekdays, whereas more frequent visits to playground were associated with less ST on weekends among children. In addition, more frequent visits to nature, own yard, and playground were associated with less whole-week ST, and higher parent–child total outdoor visit frequency was associated with less ST on weekdays and weekend days and during the whole week among children.

### Context-specific outdoor visits and weekday screen time

This study found that more frequent parent–child visits to own yard were associated with less ST on weekdays among children, suggesting that time spent in own yard might compete with time spent on screens on weekdays. A previous study in Finland showed that frequent visits to own yard were associated with less sedentary time among preschoolers on weekdays [52], supporting the findings of our study measuring sedentary ST. Own yard is close to home, and in Finland most houses and condominiums have their own yard. Therefore, own yard may be a feasible outdoor space to visit also during busy everyday life. Indeed, own yard is the most visited leisure-time outdoor space for Finnish preschool-aged children [12, 52]. However, own yards vary in size and characteristics. An Australian study found that larger yard size and greater vegetation were associated with more time spent in the yard among 2–5-year-old children [53]. Furthermore, characteristics of the house’s outdoor space seem to be associated with young children’s ST [44]. In Finland, large yards can be found especially in less populated areas, although private house and condominium yards regardless of their geographical location often also contain natural elements and play equipment, such as swings and climbing apparatus, which might make them appealing places for frequent visits on weekdays. In addition, parents’ safety concerns are a common barrier for young children’s outdoor play [34, 36], and own yard as a private, safe, and familiar space might alleviate these concerns.

Moreover, this study showed that more frequent parent–child nature visits were associated with less weekday ST among children. The result is supported by a previous finding that more frequent parent–child nature visits are associated with less weekday sedentary time among Finnish preschoolers [54]. Although the accessibility of nature, as well as definitions of nature, might vary according to the living environment and country, studies conducted in other countries have shown similar results. For example, green spaces closer to home were associated with less ST in school-aged children [54] and less TV-viewing time in preschool-aged children [45]. However, in Finland, nature is nearby in both rural and urban areas, and 50% of Finns live only 300 m away from the nearest forest [55]. In addition, in Finland, everyone’s right allows access to nature without obtaining permission from the landowner [56], which makes nature an accessible outdoor space. Hence, visiting nature is a rather common behaviour for families with young children, although the frequency of visits varies between studies [12, 33, 47].

Furthermore, more frequent parent–child total outdoor visits were associated with less child ST on weekdays. In Finland, it is not uncommon that parents take their children to and from ECEC by foot or by bike, especially if the distance is short [12], and parent–child outdoor visits, including nature and own yard visits, might occur on the way to and from daycare. Hence, varying outdoor spaces close to home and ECEC centres might promote frequent, spontaneous outdoor visits also on weekdays, providing an alternative to ST.

### Context-specific outdoor visits and weekend ST

The results of this study showed that higher parent–child playground visit frequency was associated with less weekend ST among children. A similar result was found in a study in the USA among low SES families, where higher park visit frequency on weekends was linked to less TV-viewing time among 2–4-year-olds [57]. One explanation for the result of our study could be that playgrounds were more often visited on weekends. However, in this study only the weekly frequency of outdoor visits was examined, not whether the visits occurred on weekdays or weekends. When interpreting the results regarding playground visits (‘C. Park/playground’) in this study, it should be noted that in Finnish, the concepts ‘park’ and ‘playground’ (‘*puisto*,* leikkipuisto’*) both refer to outdoor recreation spaces outside one’s own yard and are sometimes used as synonyms. However, the item’s formulation may have led to varied interpretations among respondents, as ‘park’ can also refer to a broader recreational area, whereas ‘playground’ is a more specific term designated for children’s play.

In addition, playgrounds might not have been equally accessible to all study participants in our sample, as the data were collected in communities that also include rural areas, where public playground density may be lower than in urban areas. Previous studies have shown that the use of outdoor play spaces varies according to living environment, with urban children using more parks and playgrounds and rural and small-town children playing more in their own yards [12]. In addition, in this study playgrounds were visited relatively seldom, only 3 times per month or less in 52% of the families, whereas another study in Finland showed playgrounds to be a more common place for families with young children to visit [12]. However, the difference could be explained by the study by Mehtälä et al. [12] assessing time spent in playgrounds, while our study assessed visit frequency. Altogether, these results suggest that a potential strategy for health promotion efforts aimed at reducing children’s ST could be to develop ways to attract families with young children to spend time in playgrounds more frequently during weekends.

This study found also that higher parent–child total outdoor visit frequency was connected to lower weekend ST. As weekends often provide more leisure time, children may have time for both outdoor and screen activities. Indeed, families spend time outdoors together especially on weekends [12], while at the same time young children typically also have more ST on weekends than on weekdays [12, 15]. Accordingly, in this study, children had on average over 2 h of ST on weekend days, nearly an hour more than on weekdays. Hence, the variety of different spaces visited, and the higher total outdoor visit frequency may play a bigger role in children’s weekend ST than visiting a single outdoor space.

### Context-specific outdoor visits and whole-week screen time

The results showed that more frequent visits to nature, own yard, playground, and in total were associated with less whole-week ST among children. The result is consistent with an earlier study showing associations between less outdoor play time and more ST in preschool children [7]. However, null results were found in two Canadian studies, where young children’s outdoor time was not associated with adherence to ST guidelines [22], and outdoor play or neighbourhood characteristics were not related to children’s ST [58]. Regarding the somewhat inconsistent results of previous studies, this study adds valuable evidence to the research of young children’s ST and outdoor visits.

### Strengths, limitations, and future directions

A strength of this study is that the sample represents all PEL groups (low, middle, and high), even though the participating parents were slightly more highly educated than 30–50-year-olds overall in Finland [59]. The indicators used for SES have been used to describe the Finnish families’ standing also previously in DAGIS studies (e.g [48, 52, 60]). Furthermore, the sample was collected in two municipalities with both rural and urban areas, and the analyses were controlled for season, which are important factors to acknowledge when evaluating children’s outdoor time [8, 61, 62]. In addition, ST was assessed with a diary, decreasing the risk for recall bias. Moreover, the outdoor visit frequency measure did not limit outdoor activities to a specific activity (e.g. play or physical activity) but was “open” in terms of activity, thus better capturing all possible activities and visits. A further strength is that this study measured actual visits to outdoors and not only availability of outdoor spaces. To our knowledge, this is one of the few studies investigating the frequency of context-specific outdoor visits and ST among preschool-aged children outside preschool hours.

This study also has some limitations that should be addressed. Being cross-sectional, no causality between the two behaviours can be detected. Also, the data used in this study are parent-reported, which can lead to social desirability bias. In addition, although the ST diary might decrease recall bias, there may be some overreporting of ST, as it was asked separately for different devices and summarized. It should also be considered that the data were collected 7–8 years ago, and children’s screen use has likely changed since then. In addition, including only the respondents who had data on all variables in the respective regression models may have reduced statistical power, and the results may be biased if the data are not missing completely at random.

Furthermore, some of the factors that have in previous studies been found to correlate with child ST and outdoor time, such as parental rules, perceived barriers and facilitators [32, 38, 63], parent ST [64], and characteristics of the environment [65], were not controlled in this study. Indeed, the effect sizes of outdoor visits were relatively modest, suggesting that factors not accounted for in this study may play a larger role in children’s ST than outdoor visits. However, from a broader public health perspective, even this small association is a valuable addition to the overall picture of factors shaping children’s ST.

A further aspect to consider regarding the limitations of this study is that data collection took place between September and November, a period when daylight rapidly decreases and temperatures drop in Finland. It is plausible, however, that across seasons the outdoor and ST behaviours of families vary, which might affect the examined associations. In addition, all outdoor spaces that young children visit might not have been captured here. Children also play on more informal sites, such as lawns and fields [12, 31], ditches, streets, and sidewalks, as well as other public spaces like school yards and sport fields [31], which were not enquired in the questionnaire.

The results of this study can inform future interventions aimed at reducing young children’s ST and increasing outdoor time. Future studies could benefit from more specific measurement instruments that account for variations in the examined behaviours between weekdays and weekends. Furthermore, the findings can contribute to health promotion strategies targeting children’s ST through outdoor activities. The results also offer insights for municipalities and policymakers planning residential areas, highlighting the importance of versatile outdoor spaces for children’s health behaviours—especially in the context of increasing urbanization across countries. Future longitudinal and intervention studies are needed to establish the eventual causal relationships between context-specific outdoor visits and children’s ST. In addition, more detailed examination of outdoor visits, for example, the length and timing of the visits and the type of activities, is needed to elucidate what kinds of outdoor visits associate with less child ST and to better promote outdoor visits in families with young children.

### Conclusion

The results of this study showed that context-specific and total outdoor visits associate with preschool children’s ST on weekdays and weekends. Visiting different outdoor spaces is linked to ST differently on weekdays and weekends, although spending time outdoors regardless of the type of outdoor space is associated with less whole-week ST. The results suggest that different contexts play a role in children’s ST at different times of the week and that frequent visits to versatile outdoor spaces during the week might help decrease child ST throughout the week. Hence, parents should be encouraged to engage in frequent visits to their preferred outdoor spaces. In addition, municipalities should ensure that a variety of outdoor spaces—built and unbuilt, public and private—are available at a feasible distance in neighbourhoods. It could provide alternatives to young children’s screen use for families from all SES backgrounds, promoting healthy lifestyles and children’s optimal development.

## Supplementary Information

Below is the link to the electronic supplementary material.

Supplementary Material 1

## Data Availability

The questionnaires and screen time diary used in the study are publicly available at https://dagis.fi (in Finnish). The dataset used during the current study is available from the corresponding author on reasonable request.

## Data availability

The questionnaires and screen time diary used in the study are publicly available at https://dagis.fi (in Finnish). The dataset used during the current study is available from the corresponding author on reasonable request.

## Declarations

### Clinical trial number

ISRCTN57165350, Registration date 08/01/2015.

## Abbreviations

ST: Screen time
ECEC: Early childhood education and care
