# Supplementary material for: Associations between parent–child outdoor visits and preschool-aged children’s screen time: a cross-sectional study
Source: J Act Sedentary Sleep Behav. 2025 Nov 6;4:18. doi: 10.1186/s44167-025-00089-5 (PMC12590795; doi:10.1186/s44167-025-00089-5)
Supplement: Supplementary file 1 — Supplementary Material 1 [file 44167_2025_89_MOESM1_ESM.docx]

|  | **1.** | **2.** | **3.** | **4.** | **5.** | **6.** | **7.** | **8.** | **9.** | **10.** | **11.** | **12.** | **13.** |
| --- | --- | --- | --- | --- | --- | --- | --- | --- | --- | --- | --- | --- | --- |
| **1. Weekday screen time** | ─ |  |  |  |  |  |  |  |  |  |  |  |  |
| **2. Weekend day screen time** | 0.594*** | ─ |  |  |  |  |  |  |  |  |  |  |  |
| **3. Whole-week screen time** | 0.934*** | 0.830*** | ─ |  |  |  |  |  |  |  |  |  |  |
| **4. Parent-child nature visit frequency** | -0.088* | 0.017 | -0.078 | ─ |  |  |  |  |  |  |  |  |  |
| **5. Parent-child playground visit frequency** | -0.074 | -0.104** | -0.100* | 0.152*** | ─ |  |  |  |  |  |  |  |  |
| **6. Parent-child own yard visit frequency** | -0.117** | -0.073 | -0.128*** | 0.336*** | 0.089* | ─ |  |  |  |  |  |  |  |
| **7. Total parent-child outdoor visit frequency** | -0.142*** | -0.090* | -0.0152*** | 0.674*** | 0.460*** | 0.777*** | ─ |  |  |  |  |  |  |
| **8. Child's sex** | 0.096* | 0.061 | 0.079* | 0.058 | 0.045 | 0.133*** | 0.123** | ─ |  |  |  |  |  |
| **9. Child's age** | 0.121** | 0.188*** | 0.164*** | -0.052 | -0.125*** | -0.051 | -0.108** | 0.008 | ─ |  |  |  |  |
| **10. Highest parental education level in the family** | -0.163*** | -0.102* | -0.169*** | 0.051 | -0.023 | 0.044 | 0.043 | -0.032 | 0.016 | ─ |  |  |  |
| **11. Relative household income** | -0.094* | -0.071 | -0.111** | -0.021 | -0.072 | 0.099* | 0.015 | 0.068 | 0.066 | 0.371*** | ─ |  |  |
| **12. Response month** | 0.067 | 0.064 | 0.079 | -0.005 | -0.165*** | -0.023 | -0.060 | -0.024 | 0.052 | 0.018 | -0.001 | ─ |  |
| **13. Respondent** | 0.056 | -0.036 | 0.037 | 0.015 | 0.091* | -0.019 | 0.039 | -0.028 | -0.060 | 0.003 | -0.038 | 0.024 | ─ |

**Supplementary material 1. Spearman’s correlations between the study variables (n=673).**

*** p<0.001, **p<0.01, *p<0.05
